# Delayed implant rehabilitation following iliac crest grafting for traumatic dentoalveolar defects: a prospective single-arm interventional clinical study

**DOI:** 10.1186/s12903-026-08857-8

**Published:** 2026-06-19

**Authors:** Mohamed Abdeldayem, Mariam Ahmed Raafat Abd EL Hamid Mohamed, Shady Aly Hassan, Naglaa Shokry Abdelsalam

**Affiliations:** 1 Faculty of Dentistry, CranioMaxilloFacial and Plastic Surgery Department, Alexandria University, Champollion St., Azarita, Alexandria, 21527 Egypt; 2 Faculty of Dentistry, Oral and Maxillofacial Surgery Department, Suez University, Suez, Egypt

**Keywords:** Iliac Bone Graft, Delayed alveolar reconstruction, Dental implants, Post-traumatic defects, Implant stability

## Abstract

**Background:**

Implant rehabilitation following post-traumatic dentoalveolar defects can be challenging due to irregular bone loss and insufficient ridge volume that compromise dental implant placement and prosthetic rehabilitation.

**Aim of the study:**

To evaluate implant rehabilitation following reconstruction of delayed post-traumatic maxillary and mandibular dentoalveolar defects using iliac crest bone grafts in terms of implant stability (primary outcome), marginal bone loss, graft success, and patient satisfaction (secondary outcomes).

**Methods:**

This prospective interventional single-arm clinical study included 20 patients (18–65 years), both sexes presenting with post-traumatic alveolar bone defects who underwent reconstruction using anterior iliac crest grafts, followed by implant placement after 6 months and prosthetic rehabilitation. Post-operative assessments primarily evaluated implant stability and secondarily assessed marginal bone loss, graft success, and patient satisfaction. All adverse events were recorded during follow-up.

**Results:**

Graft associated with improved implant stability significantly from 59.7 ±10.01 Ncm at baseline to 68.7 ±10.17 Ncm after 6 months (P < 0.001). Horizontal graft width increased from 2.1±0.5 mm preoperatively to 6.9±0.8 mm at 6 months (P<0.001), while vertical graft height improved from 7.2±1.1 mm to 10.7±1.3 mm (P<0.001). Median marginal bone loss reached 1.5 mm at 6 months (P < 0.001). A total of 30 implants were placed with an implant survival rate of 96.7% (29/30). Graft success was achieved in all cases. Most patients reported high satisfaction, with 85% being very satisfied or satisfied. A single case of donor site wound dehiscence was reported.

**Conclusions:**

Iliac crest bone grafting may represent a clinically promising approach for reconstruction of post-traumatic dentoalveolar defects, allowing favorable short-term implant stability following delayed implant rehabilitation.

**Trial registration:**

The research was registered retrospectively in pactr.samrc.ac.za (PACTR202511489819034) on 11-11-2025.

## Introduction

Reconstruction of post-traumatic maxillofacial bone defects remains a major challenge in oral and maxillofacial surgery (OMFS) [1]. Loss of dentoalveolar bone following trauma often results in functional and aesthetic problems, making alveolar ridge reconstruction critical for jaw integrity and successful dental implant rehabilitation [2].

Dental implants have become the standard approach for rehabilitating partial or complete edentulism [3]. However, adequate bone volume and quality are crucial for implant stability and long-term success. In many trauma cases, bone defects resulting from fractures or dentoalveolar injuries limit implant placement, necessitating prior alveolar ridge reconstruction [4, 5].

Autologous bone is considered the gold standard due to its osteogenic, osteoconductive and osteoinductive properties. Among available donor sites, the anterior iliac crest (AIC) provides sufficient cortical and cancellous bone for extensive reconstructions of maxillofacial defects [6–9].

A two-stage approach is typically employed, with 4–6 months for graft consolidation before implant insertion [10]. Graft integration is monitored clinically and radiographically, ensuring proper vascularization and minimizing risks of micromotion and infection. This method enhances implant survival and facilitates optimal prosthetic and soft-tissue outcomes [11].

Unlike elective ridge augmentation in atrophic jaws, post-traumatic jaw defects are often more complex due to complications like irregular bone loss patterns, compromised soft tissue vascularity, extensive scarring and soft-tissue dehiscence [12].

Although iliac crest bone grafting is widely used for alveolar reconstruction, the available literature evaluating delayed implant rehabilitation following traumatic dentoalveolar injuries remains limited, with most published studies restricted to isolated case reports. Consequently, evidence regarding graft integration, implant survival, and functional rehabilitation outcomes in trauma-related dentoalveolar defects remains scarce, highlighting the need for prospective clinical studies in this field [13]. 

This study aimed to evaluate implant stability, marginal bone loss, graft success in terms of graft integration and adequate bone formation for implant placement, implant survival, and patient satisfaction following two-stage reconstruction of post-traumatic dentoalveolar defects using anterior iliac crest bone grafts prior to delayed implant rehabilitation.

The null hypothesis was that iliac crest bone graft augmentation would not result in significant improvement in alveolar ridge dimensions or implant stability, and would not achieve satisfactory implant survival or patient-reported outcomes.

## Materials and methods

Patients with post-traumatic segmental alveolar defects of the maxilla or mandible indicated for surgery were recruited prospectively between August 2024 and April 2025. All cases were admitted, operated on and followed up at CranioMaxilloFacial and Plastic Surgery Department, Faculty of Dentistry, Alexandria University, Alexandria, Egypt.

The primary outcome was implant stability, while the secondary outcomes included marginal bone loss, graft success and patient satisfaction. The study protocol was established prior to patient enrollment and all reported outcomes adhered to the original study protocol and were not modified after patient recruitment.

Sample size was based on Rosner’s method [14] calculated by G*Power 3.1.9.7 [15]. Sample size estimation was conducted, assuming a 95% confidence level, 5% alpha error, and 80% study power. According to Kniha et al. [7], implant stability was 60.93 and it was estimated to increase to 69.01 after 6 months. Based on a comparison of dependent means and SD of 8.09, a minimum of 18 patients was required to detect an effect size of 0.707. To account for potential loss to follow up, this number increased to 20 patients. Implant stability outcomes were analyzed at the implant level, whereas sample size calculation was performed at the patient level.

Inclusion criteria included adults (18–65 years), of both genders, absence of medical conditions contraindicating iliac bone grafting surgery and implant surgery, who required reconstruction of traumatic alveolar bone loss. These injuries led to delayed bone defects characterized by insufficient volume for implant placement or achievement of implant stability (Defects were standardized as Class IV or Class V according to Cawood and Howell ridge classification) [16]. Patients with uncontrolled systemic diseases such as Diabetes mellitus, heavy smokers (> 10 cigarettes/day), untreated periodontitis or gingivitis, severe bruxism, clenching habits or prior failed bone grafting in the same site were excluded.

### Ethical compliance and registration

The study was conducted in accordance with the Helsinki Declaration (2013) and approved by the research ethical committee of Faculty of Dentistry, Alexandria University Hospitals (IRB No. 001056 – IORG 0008839) (approval code: 1139-08/2025) and retrospectively registered on pactr.samrc.ac.za (PACTR202511489819034) on (11-11-2025). Retrospective registration occurred due to administrative and institutional procedures. Informed consent was obtained from each patient prior to their enrolment and any surgical intervention after explaining the study objectives and procedures.

### Blinding and bias mitigation

Data collection and radiographic assessment were performed by an independent investigator to reduce assessment bias. All radiographic measurements were performed twice by the same examiner with a two-week interval between measurements, and the mean values were used for analysis to improve measurement reliability. Excellent intra-examiner reliability was achieved, with ICC values ≥ 0.99 and narrow confidence intervals close to 1.0.

*Treatment protocol included two phases as follows: 

### Presurgical phase 

All participants underwent a comprehensive preoperative evaluation as detailed history, clinical and radiographical examination including:

- Extraoral evaluation (focusing aesthetic concerns)
- Intraoral examination to evaluate oral hygiene and dental status
- Cone-beam computed tomography (CBCT) was performed to assess bone availability and reveal defects in the maxilla or mandible insufficient for implant placement.

### Surgical procedure

TS approach was adopted (initial harvesting of an alveolar bone graft (ABG) from the AIC, followed by implant placement).

#### First surgical stage

1. All patients received preoperative prophylactic antibiotic and were treated under general anaesthesia.
2. At donor site, a monocortical ABG, including the medial cortex and cancellous portion, was harvested.
3. At the recipient site, a full thickness mucoperiosteal flap was elevated and the bed prepared. Periosteal releasing incisions were performed when necessary to achieve adequate flap advancement and tension-free primary closure over the grafted site. Careful soft tissue handling and layered closure were performed to minimize the risk of wound dehiscence or graft exposure. No adjunctive soft tissue augmentation procedures were performed. Despite the traumatic nature of the defects, adequate soft tissue coverage was achieved in all cases through careful flap advancement and tension-free closure.
4. The ABG was shaped, secured with titanium mini-screws (cancellous surface facing native bone), slightly oversized to compensate for resorption, and gaps filled with additional cancellous bone from the donor site.

#### Second surgical stage

After six months, CBCT confirmed graft integration. A total of 30 dental implants were placed in the augmented sites using the Neodent Implant System. The implants were standardized across all patients to ensure reproducibility of the surgical outcomes. Following six months of osseointegration, implants were exposed and restored with fixed prostheses.

##### Prosthetic rehabilitation and post-loading care

**Fig. 1:**
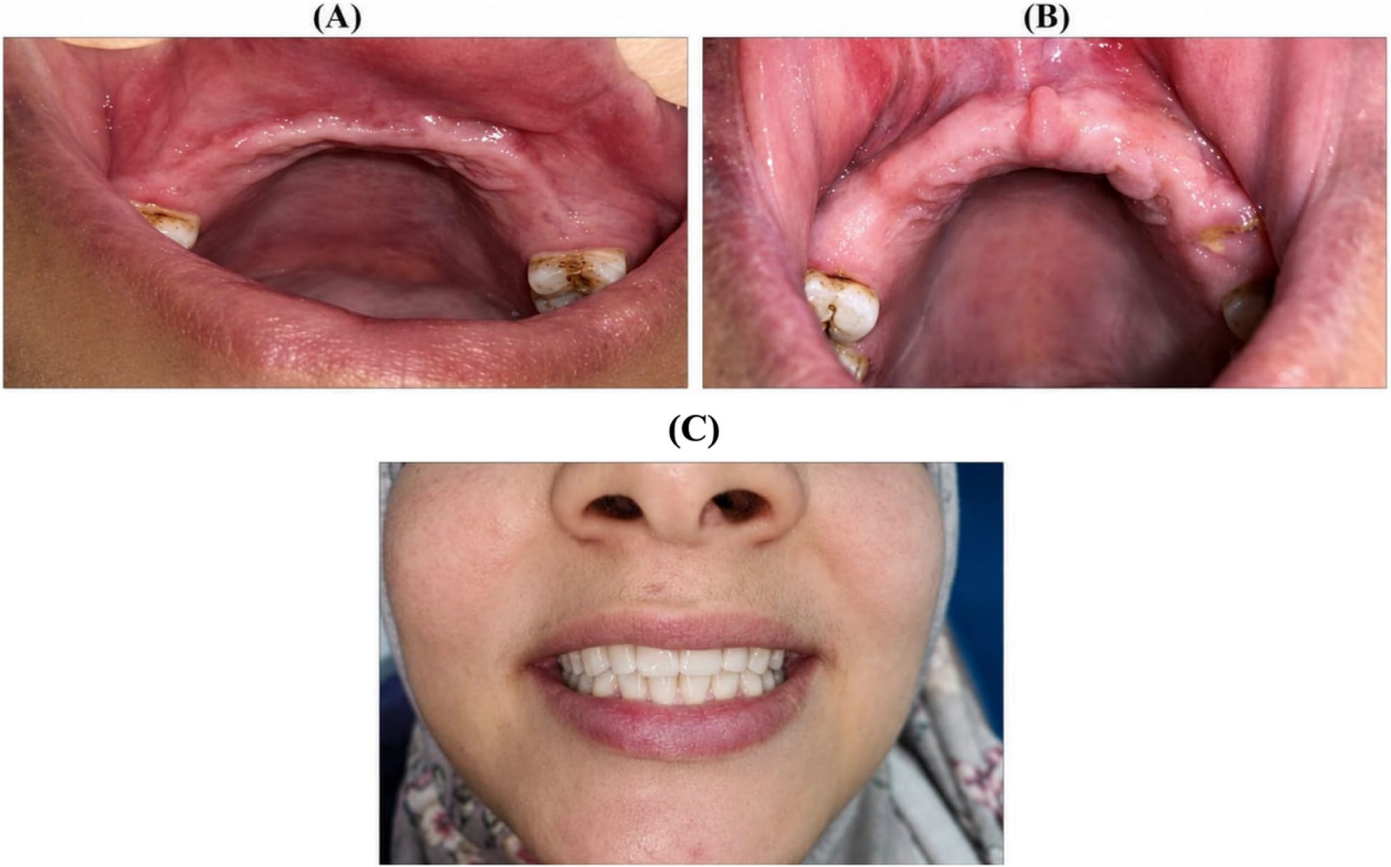
Clinical photographs of delayed implant rehabilitation following iliac crest graft augmentation. **A** Preoperative clinical view showing traumatic alveolar ridge defect. **B** Clinical appearance after alveolar ridge augmentation demonstrating improved ridge contour and width. **C** Final prosthetic rehabilitation with satisfactory functional and aesthetic outcome

Six months post-implant placement, final prosthetic rehabilitation was performed. Patients received Screw retained bridges and crowns with zirconia superstructure. Post-loading care included a standardized oral hygiene protocol with adequate cleaning and radiographic assessment every 3 months during the first year (Fig. 1).

CBCT Acquisition: All CBCT scans were performed using the same device (Planmeca ProMax 3D, Planmeca, Helsinki, Finland) at baseline and 6 months post-operatively. To ensure measurement reliability, the imaging parameters were standardized for all patients: tube voltage 90 kVp, tube current 12 mA, and a voxel size of 0.2 mm. The Field of View (FOV) was standardized to encompass the entire surgical site while maintaining high resolution. All volumetric data and linear measurements of bone loss were analysed using (Planmeca Romexis software, version 4.4.3). Standardized anatomical landmarks were used in all CBCT measurements to ensure reproducibility.

*Follow up phase included standardized postoperative intervals including immediate postoperative assessment, 1 week, 1 month, 3 months and 6 months after implant placement to assess the following parameters:

1. Patient satisfaction was evaluated using a five-point Likert scale (1 = very dissatisfied, 2 = dissatisfied, 3 = neutral, 4 = satisfied and 5 = very satisfied) [17].
2. Graft success was assessed based on the absence of graft exposure during healing, infection, radiolucency on routine radiographic examination, graft mobility at the recipient site, and failure to achieve adequate bone formation for implant placement. Intraoperative bleeding from the graft during implant drilling was considered a clinical indicator of graft integration and vitality. Graft success criteria were adapted from previously published clinical and radiographic assessment protocols [18].
3. Implant stability was assessed using a calibrated electronic surgical motor (NSK Surgic Pro). Implant stability was assessed at the time of implant placement and after a 6-month healing period prior to prosthetic loading to evaluate osseointegration [19]. Implant success was defined by the absence of implant mobility, persistent pain, infection, or implant loss during follow-up.
4. Bone Loss: Immediately post-grafting and after 6 months CBCT assessment was performed to evaluate the bone height changes in grafted sites as following: In maxilla, the upper limits of bone graft are nasal floor and the maxillary sinus floor that determined by connection of 4 points (1st at the lowest part of the nasal floor, 2nd and 3rd at the lower parts of the mesial and distal walls and 4th is the median point between points 2nd and 3rd points) [16, 20]. In mandible, distance between the alveolar ridge and the border of the jaw was recorded [21].

All adverse events were recorded prospectively during follow-up visits, including infection, wound dehiscence and any radiation-related issues due to repeat imaging.

* Statistical analysis was done by SPSS v29 (IBM Inc., Chicago, IL, USA). Shapiro-Wilks’s test and histograms were used to evaluate the normality of the data distribution. Quantitative variables were presented as mean and standard deviation (SD) and compared between the measurements utilizing paired t- test. Quantitative non-parametric data were presented as median and interquartile range (IQR) and were compared by Wilcoxon test. Qualitative variables were presented as frequency (%) and were analyzed utilizing McNemar test. A two tailed *P* < 0.05 was considered statistically significant.

## Results

There were 8 (40%) males and 12 (60%) females with mean age of 46.6 (10.57). All patients presented with post-traumatic bone loss, categorized as Class IV (45%) and Class V (55%) alveolar ridge types Table 1.

**Table 1:** Demographic data of the studied patients

Variables		Findings (*N* = 20)
Age (years), Mean (SD)		46.6 ± 10.57
Sex, n (%)	Male	8 (40%)
	Female	12 (60%)
Site of defect, n (%)	Maxilla	12 (60%)
	Mandible	8 (40%)
Cawood and Howell classification, n (%)	Class IV (knife-edge)	9 (45%)
	Class V (flat)	11 (55%)

SD: standard deviation, n: frequency, %: percentage

**Fig. 2:**
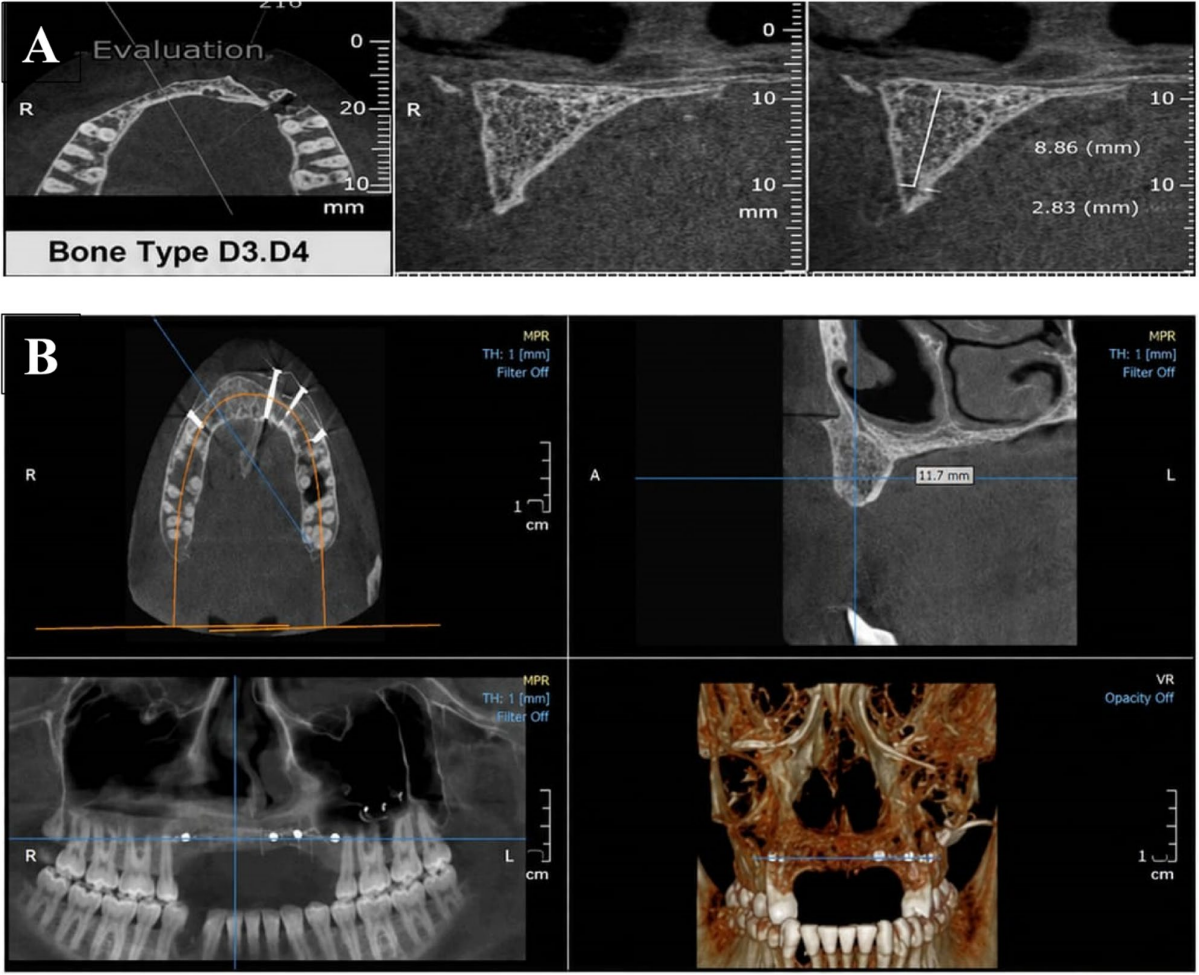
CBCT radiographic assessment of alveolar ridge augmentation using iliac crest bone grafting. **A** Preoperative CBCT images demonstrating traumatic alveolar ridge deficiency with reduced ridge width and inadequate bone volume for implant placement. **B** Postoperative CBCT images after graft consolidation showing increased alveolar ridge thickness and improved bone volume prior to delayed implant placement

Radiographical follow-up showed a significant increase in alveolar ridge dimensions at the 6-month follow-up interval. The mean horizontal bone width increased from 2.1 ± 0.5 mm at baseline to 6.9 ± 0.8 mm (*P* < 0.001), while the mean vertical bone height improved from 7.2 ± 1.1 mm to 10.7 ± 1.3 mm (*P* < 0.001) (Figs. 2A and B).

Graft associated with improved implant stability significantly after 6 months (68.7 ± 10.17 Ncm) than baseline (59.7 ± 10.01 Ncm) (*P* < 0.001). Bone loss was significantly higher after 6 months [1.5 (0–3) mm] compared with baseline [0 (0–0) mm] (*P* < 0.001) Table 2.

**Table 2:** Implant stability and bone loss of the studied patients

Variables	Baseline	After 6 months	*P*-value	MD (95% CI)
Horizontal bone width (mm)	2.1 ± 0.5	6.9 ± 0.8	< 0.001*	4.8 (4.37: 5.23)
Vertical bone height (mm)	7.2 ± 1.1	10.7 ± 1.3	< 0.001*	3.5 (2.73: 4.27)
Implant stability (Ncm), Mean (SD)	59.7 ± 10.01	68.7 ± 10.17	< 0.001*	9 (2.54: 15.46)
Bone loss (mm), Median (Range) (After implant placement)	0 (0–0)	1.5 (0–3)	< 0.001*	1 (0:2)

*Ncm* Newton centimeter, *mm* Millimeter, *SD* Standard Deviation, *MD* Mean difference or median difference, *CI* Confidence interval. Data are presented as mean ± SD for normally distributed variables and median (range) for non-normally distributed variables. *Statistically significant at P < 0.05 using paired t-test or Wilcoxon signed-rank test.

At the second-stage surgery (reopening), a mean graft resorption of 1.1 mm was recorded; however, this did not appear to compromise implant placement or the planned rehabilitation protocol Table 3.

**Table 3:** Graft Resorption Before Implant Placement

Variables	Baseline	After 6 months
Graft Resorption Before implant Placement (mm), Mean ± SD	-	1.1 ± 0.3

*mm* Millimeter, *SD* Standard Deviation, *MD* Mean difference or median difference, *CI* Confidence interval

Regarding bone grafting success rate demonstrated 100% in terms of graft integration and immobilization before implant placement, no graft exposure, no persistent infection, or peri-graft radiolucency were observed during the 6-month healing period. During implant placement, all grafted sites exhibited healthy bleeding during osteotomy preparation, suggesting satisfactory graft vitality and vascularization for implant insertion.

The implant survival rate was 96.7%, with 29 out of 30 implants remaining clinically stable during follow-up. One implant failure (3.3%) was recorded and managed accordingly. Regarding patient satisfaction, 13 patients (65%) were very satisfied, 4 (20%) were satisfied, 2 (10%) were neutral, and 1 (5%) was dissatisfied Table 4.

**Table 4:** Success rate and patient satisfaction of the studied patients

Variables		Findings (*N* = 20)
Graft success outcomes	Successful integration & Immobilization	20 (100%)
	Absence of Exposure/Infection	20 (100%)
	Bleeding during drilling	20 (100%)
	Adequate bone formation	20 (100%)
Implant outcomes	Total number of implants placed	30
	Successful implants	29
	Implant survival rate	96.7%
	Failure rate	3.3%
Patient satisfaction	Very satisfied	13 (65%)
	Satisfied	4 (20%)
	Neutral	2 (10%)
	Dissatisfied	1 (5%)
	Very dissatisfied	0 (0%)

Data are presented as frequency (%)

Regarding postoperative complications, one patient (5%) experienced wound dehiscence at the donor site (iliac crest) during the first week. This was managed promptly through adequate debridement, resuturing under local anesthesia, and extended antibiotic coverage. The site healed completely without further complications or long-term morbidity. All other patients showed uneventful healing at both donor and recipient sites. No clinically significant vestibular obliteration was observed, and vestibuloplasty was not required during the second-stage procedure.

## Discussion

Graft consolidation was associated with significant improvement in implant stability. Mean insertion torque increased from 59.7 ± 10.01 Ncm at baseline to 68.7 ± 10.17 Ncm after six months. Moderate alveolar bone resorption occurred during the healing period. Radiographically, median crestal bone loss was 1.5 mm (IQR 0–3.0) after six months. A high implant survival rate 96.7% (29 out of 30 implants) was observed, with favorable patient satisfaction, as 85% of patients reported being satisfied or very satisfied with the final functional and aesthetic outcomes.

The increase in stability likely reflects new bone formation and densification in the augmented ridge. Similarly, Ivanova et al. [22] found a strong positive correlation between implant stability and both bone density and percentage of vital bone formation. As the iliac graft remodelled and incorporated, the bone–implant interface strengthened. This is consistent with established principles of two-stage augmentation: a healing period of at least 4–6 months usually ensures sufficient bone maturation to support high implant stability [23].

Vallecillo-Rivas et al. [13] reported that implants placed in regenerated bone achieved acceptable stability by 12 weeks. However, stability values remained lower than those observed in native bone unless a sufficient healing interval was allowed. Our protocol of a six-month consolidation before implant placement may have contributed to the favorable stability outcomes observed in this single-arm prospective interventional clinical study.

Moderate alveolar bone resorption reflects the well-documented remodeling process of onlay bone grafts. Some degree of crestal bone remodeling is expected during the early healing phase as the graft undergoes revascularization and biological adaptation. Therefore, the observed 1.5 mm bone loss at 6 months should be interpreted as an early remodeling change rather than a predictor of long-term marginal bone stability.

Also, Attar et al. [24] reported favorable implant-related outcomes in iliac crest grafts, including reduced bone loss and complete osseointegration without implant failures. Likewise, Shirani et al. [25] observed acceptable implant survival, aesthetic satisfaction, and moderate graft resorption during follow-up [26]. Even over longer follow-up periods, implant survival in iliac-augmented sites remained high (often ≥ 90%) as reported by Beck et al. [10].

This biological response may be explained by functional bone remodeling under occlusal loading, consistent with Wolff’s Law, where the iliac graft may undergo adaptive remodeling during functional loading [27].

Although poor oral hygiene and inadequate compliance with postoperative maintenance protocols may have contributed to implant failure, other factors such as graft remodeling, local bone quality, and surgical variables cannot be completely excluded. The failed implant site occurred in the maxilla. Importantly, the underlying iliac bone graft at the failure site remained clinically stable and well-integrated during follow-up, suggesting that graft instability was less likely to be the primary cause of failure.

Similar to our observations, Beck et al. [10] noted a higher failure risk than native bone (6 vs. 0 failures), but still only 8% of implants were lost over about 6 years. The favorable short-term implant survival observed in the present study may be related to careful case selection and the adopted two-stage approach.

Patient satisfaction in our study was comparable to previous reports. Gjerde et al. [8] reported 90.5% overall satisfaction after iliac crest reconstruction, while Wortmann et al. [9] found no significant difference in satisfaction between iliac and calvarial donor sites. However, McKenna et al. [28] reported higher survival rates with intraoral donor sites. In the present series, large traumatic segmental defects required greater graft volume, for which intraoral grafts were insufficient. Although iliac grafts may show slightly higher resorption, they remain a practical option when substantial bone volume is required. Wortmann et al. [9] also noted early gait disturbance associated with iliac donor sites, a morbidity minimized in the present study through careful surgical technique and postoperative counseling.

The only notable complication was donor site wound dehiscence in one patient, which was successfully managed with debridement and resuturing, leading to healing without affecting the final outcome.

Although several outcomes demonstrated statistical significance, their clinical relevance should be interpreted within the context of the relatively short follow-up period and single-arm study design.

The six-month follow-up interval was selected because it corresponds to the commonly accepted consolidation period before delayed implant placement following iliac crest graft reconstruction. This time point allows assessment of early graft integration and implant stability, which represented the predefined clinical outcomes of the present study. In addition, it reflects the clinical decision-making stage at which surgeons determine the suitability of the grafted site for implant placement and prosthetic rehabilitation.

The present study has several limitations. First, the relatively small sample size, absence of a control group, and short follow-up duration restrict direct comparison with alternative grafting techniques and limit definitive conclusions regarding comparative efficacy and long-term outcomes. The present findings primarily reflect early healing and graft integration rather than long-term implant survival, graft stability, or functional loading outcomes. In addition, iliac crest grafts are known to undergo continued remodeling beyond 6–12 months, which could not be evaluated within the current follow-up period. Therefore, the present findings should be interpreted as preliminary short-term feasibility data rather than evidence of long-term clinical performance.

Second, implant-level analysis without adjustment for clustering may have reduced statistical independence between observations, and subgroup analysis between maxillary and mandibular sites was not performed due to the limited sample size.

Third, no formal adjustment for multiple comparisons was performed because of the exploratory nature and limited sample size of the study. Furthermore, graft vitality was assessed radiographically rather than histologically, and patient satisfaction was evaluated using a simple Likert scale, which may limit the depth of outcome assessment.

Finally, implant stability was assessed using insertion torque values without resonance frequency analysis (RFA), and retrospective trial registration represents an additional methodological limitation. Moreover, exclusion of high-risk patients may limit generalizability of the findings to broader patient populations.

## Conclusion

Two-stage reconstruction of delayed post-traumatic maxillary and mandibular alveolar defects using iliac crest bone grafts followed by delayed implant placement demonstrated favorable short-term implant stability, graft integration, and patient-reported satisfaction during the early healing phase. These findings provide preliminary clinical feasibility data; however, long-term prospective follow-up studies are still required to evaluate implant survival, graft stability, and remodeling outcomes following functional loading.

## Data Availability

The datasets generated and analyzed during the current study are available from the corresponding author upon reasonable request.

## Abbreviations

AIC: Anterior iliac crest
CBCT: Cone-beam computed tomography
ABG: Alveolar bone graft
IQR: Interquartile range
OMFS: Oral and maxillofacial surgery
SD: Standard deviation
SWT: Sub-crestal window technique
TC: Trephine curettage
TT: Trapdoor technique
NCm: Newton Centimeter
mm: Millimeter
RFA: Resonance frequency analysis

## References

[1] Lim HK, Choi YJ, Choi WC, Song IS, Lee UL. Reconstruction of maxillofacial bone defects using patient-specific long-lasting titanium implants. Sci Rep. 2022;12:7538–985. 35534499 10.1038/s41598-022-11200-0 PMC9085892

[2] Rana M, Buchbinder D, Aniceto GS, Mast G. Patient-specific solutions for cranial, midface, and mandible reconstruction following ablative surgery: Expert opinion and a consensus on the guidelines and workflow. Craniomax Traum Rec. 2025;18:15–52. 10.3390/cmtr18010015 PMC11995815 40271473

[3] Velasco-Ortega E, Del Rocío Jiménez-Martin I, Moreno-Muñoz J, Núñez-Márquez E, Rondón-Romero JL, Cabanillas-Balsera D, et al. Long-term treatment outcomes of implant prostheses in partially and totally edentulous patients. Mater (Basel). 2022;15:856–987. 10.3390/ma15144910 PMC9316310 35888378

[4] Filipov I, Chirila L, Cristache CM. Rehabilitation of extremely atrophic edentulous mandible in elderly patients with associated comorbidities: a case report and proof of concept. Head Face Med. 2021;17:22–65. 34187501 10.1186/s13005-021-00274-2 PMC8240274

[5] Alshamrani AM, Mubarki M, Alsager AS, Alsharif HK, AlHumaidan SA, Al-Omar A. Maxillary sinus lift procedures: An overview of current techniques, presurgical evaluation, and complications. Cureus. 2023;15:495–539. 10.7759/cureus.49553 PMC10753870 38156177

[6] Schmidt AH. Autologous bone graft: Is it still the gold standard? Injury. 2021;52:18–22. 10.1016/j.injury.2021.01.043 33563416

[7] Kniha K, Alhares J, Möhlhenrich SC, Katz MS, Winnand P, Hölzle F, et al. Dental Implant Placement in the Maxilla Following Ridge Augmentation with Free Iliac Bone Graft and Oral Rehabilitation with Fixed Prosthesis: a Three-Year Follow-Up Study. J Oral Maxillofac Res. 2024;15:3–18. 10.5037/jomr.2024.15103 PMC11131376 38812951

[8] Gjerde C, Shanbhag S, Neppelberg E, Mustafa K, Gjengedal H. Patient experience following iliac crest-derived alveolar bone grafting and implant placement. Int J Implant Dent. 2020;6:4–12. 32020348 10.1186/s40729-019-0200-8 PMC7000591

[9] Wortmann DE, van Minnen B, Delli K, Schortinghuis J, Raghoebar GM, Vissink A. Harvesting anterior iliac crest or calvarial bone grafts to augment severely resorbed edentulous jaws: a systematic review and meta-analysis of patient-reported outcomes. Int J Oral Maxillofac Surg. 2023;52:481–94. 36243645 10.1016/j.ijom.2022.09.002

[10] Beck F, Watzak G, Lettner S, Gahleitner A, Gruber R, Dvorak G, et al. Retrospective Evaluation of Implants Placed in Iliac Crest Autografts and Pristine Bone. J Clin Med. 2022;11:1367. 35268457 10.3390/jcm11051367 PMC8910966

[11] Ucer C, Khan RS. Extraction socket augmentation with autologous platelet-rich fibrin (PRF): The rationale for socket augmentation. Dent J (Basel). 2023;11:586–957. 10.3390/dj11080196 PMC10453157 37623292

[12] Thoma DS, Gil A, Hämmerle CHF, Jung RE. Management and prevention of soft tissue complications in implant dentistry. Periodontol 2000. 2022;88:116 – 29. 10.1111/prd.12415 PMC9306802 35103320

[13] Vallecillo-Rivas M, Reyes-Botella C, Vallecillo C, Lisbona-González MJ, Vallecillo-Capilla M, Olmedo-Gaya MV. Comparison of Implant Stability between Regenerated and Non-Regenerated Bone. A Prospective Cohort Study. J Clin Med. 2021;10:3220. 34362004 10.3390/jcm10153220 PMC8347999

[14] Rosner B. Fundamentals of biostatistics: Nelson Education. Nelson Educ. 2015;41:651–745.

[15] Kang H. Sample size determination and power analysis using the G* Power software. J Educ Eval Health Prof. 2021;18:651–745. 10.3352/jeehp.2021.18.17 PMC8441096 34325496

[16] Cawood JI, Howell RA. A classification of the edentulous jaws. Int J Oral Maxillofac Surg. 1988;17(4):232–6. 3139793 10.1016/s0901-5027(88)80047-x

[17] Belay Bizuneh Y, Fitiwi Lema G, Yilkal Fentie D, Woldegerima Berhe Y, Enyew Ashagrie H. Assessment of patient’s satisfaction and associated factors regarding postoperative pain management at the university of gondar compressive specialized hospital, northwest ethiopia. Pain Res Manag. 2020;2020:883–980. 10.1155/2020/8834807 PMC7676941 33273994

[18] Barone A, Covani U. Maxillary alveolar ridge reconstruction with nonvascularized autogenous block bone: clinical results. J Oral Maxillofac Surg. 2007;65(10):2039–46. 17884536 10.1016/j.joms.2007.05.017

[19] Meredith N. Assessment of implant stability as a prognostic determinant. Int J Prosthodont. 1998;11(5):491–501. 9922740

[20] Carinci F, Farina A, Zanetti U, Vinci R, Negrini S, Calura G, Laino G, Piattelli A. Alveolar ridge augmentation: a comparative longitudinal study between calvaria and iliac crest bone grafrs. J Oral Implantol. 2005;31(1):39–45. 15751387 10.1563/0-716a.1

[21] Nguyen TTH, Eo MY, Kuk TS, et al. Rehabilitation of atrophic jaw using iliac onlay bone graft combined with dental implants. Int J Implant Dent. 2019;5:11. 10.1186/s40729-019-0163-9. 30887237 10.1186/s40729-019-0163-9 PMC6423193

[22] Ivanova V, Chenchev I, Zlatev S, Mijiritsky E. Correlation between Primary, Secondary Stability, Bone Density, Percentage of Vital Bone Formation and Implant Size. Int J Environ Res Public Health. 2021;18:6994. 34208849 10.3390/ijerph18136994 PMC8297224

[23] Caggiano M, Acerra A. Implant Stability in Regenerated Bone. Appl Sci. 2023;13:12161.

[24] Attar BM, Jokar S, Naghdi N, Ghassemi A. The Success Rate of Implants Placed in Reconstructed Alveolar Ridges Using Iliac Bone Graft Compared to Non-augmented Jaw. Br J Med Med Res. 2015;9:1–7.

[25] Shirani G, Hashemi Nasab M, Bashiri S, Kordi S. Survival Rate and Cervical Bone Loss of Dental Implants Placed in Regenerated Areas with Free Iliac Graft. J Dent (Shiraz). 2023;24:53–9. 36864991 10.30476/DENTJODS.2022.90184.1474 PMC9971605

[26] Acerra A, Caggiano M, Aliberti A, Langone M, Giordano F. Early Clinical Outcomes of Full-Arch Rehabilitations with Immediately Loaded Implants with Buccal Dehiscence Treated with Horizontal Augmentation: A 1-Year Retrospective Case Series. Dent J (Basel). 2026;14(2):121. 10.3390/dj14020121 . PMID: 41744959; PMCID: PMC12939081. 41744959 10.3390/dj14020121 PMC12939081

[27] Frost HM. Skeletal structural adaptations to mechanical usage (SATMU): 1. Redefining Wolff’s law: the bone modeling problem. Anat Rec. 1990;226(4):403 – 13. 10.1002/ar.1092260402. PMID: 2184695. 10.1002/ar.1092260402 2184695

[28] McKenna G, Gjengedal H, Harkin J, Holland N, Moore C, Srinivasan M. Effect of autogenous bone graft site on dental implant survival and donor site complications: a systematic review and meta-analysis. J Evid Based Dent Pract. 2022;22:101731. 36162883 10.1016/j.jebdp.2022.101731

